# Diagnostics Receives First Impact Factor

**DOI:** 10.3390/diagnostics9020064

**Published:** 2019-06-21

**Authors:** Andreas Kjaer

**Affiliations:** Editor-in-Chief of Diagnostics. Department of Clinical Physiology, Nuclear Medicine & PET and Cluster for Molecular Imaging, Rigshospitalet and University of Copenhagen, Blegdamsvej 9, DK-2100 Copenhagen, Denmark; akjaer@sund.ku.dk; Tel.: +45-3545-4216

It is with great pleasure we can announce that *Diagnostics* has received its first official impact factor, which has just been published in the 2018 edition of the Journal Citation Reports®. The impact factor of 2.489 ranks *Diagnostics* as number 46 of 160 journals within the category “Medicine, General & Internal”; i.e., in the top 30% of the category.

The coverage by the flagship citation index Science Citation Index Expanded by Clarivate Analytics and now the release of the first official impact factor represents another major milestone for our journal following indexing and full coverage by PubMed in 2016 [1]. We believe this will further increase the visibility of *Diagnostics* in the scientific community.

The selection of a journal for coverage in the Science Citation Index Expanded includes an evaluation of basic publishing standards, editorial content, international focus and citation analysis. Therefore, we are particularly proud that *Diagnostics* is now being covered, as the evaluation criteria fit well with areas of high priority at our journal.

*Diagnostics* continues to develop in a positive way with an ever-increasing number of submissions, reaching a total of 123 in 2018, and 130 submissions were received by June 2019. Submissions are received from all over the world. A total of 85 papers were published in 2018, of which 56.5% were full-length research articles, 36.5% were reviews and the remaining were case reports or interesting images. Since the introduction of interesting images in 2015 [2], this has remained a popular category both among authors but also among clinicians, who use it as a valuable source of information for evaluating rare or difficult imaging cases.

The total number of downloads of *Diagnostics* papers from our website reached an impressive 261,381 in 2018.

All this would not have been possible without continuous support from the scientific community through submissions of high-quality papers and the extraordinary work performed by the editorial board, editorial office staff and the publisher. Thank you to everyone who has contributed.

## References

[1] Kjaer A. (2016). Diagnostics Now in PubMed and PubMed Central. Diagnostics.

[2] Kjaer A. (2015). Introducing Interesting Images. Diagnostics.

